# Investigational treatment suspension and enhanced cell-mediated immunity at rebound followed by drug-free remission of simian AIDS

**DOI:** 10.1186/1742-4690-10-71

**Published:** 2013-07-16

**Authors:** Iart Luca Shytaj, Barbara Chirullo, Wendeline Wagner, Maria G Ferrari, Rossella Sgarbanti, Alessandro Della Corte, Celia LaBranche, Lucia Lopalco, Anna Teresa Palamara, David Montefiori, Mark G Lewis, Enrico Garaci, Andrea Savarino

**Affiliations:** 1 Istituto Superiore di Sanità, Viale Regina Elena, 299, 00161, Rome, Italy; 2 BIOQUAL, Inc., 9600 Medical Center Drive, Rockville, MD 20850, USA; 3 ABL, Rockville, MD 20850, USA; 4 Università Telematica San Raffaele, via di val Cannuta 247, Rome, Italy; 5 Duke University, Durham, North Carolina, USA; 6 San Raffaele Scientific Institute, Milan, Italy; 7 Department of Public Health and Infectious Diseases, Institute Pasteur, Cenci-Bolognetti Foundation, “Sapienza”, University of Rome, Rome, Italy; 8 San Raffaele Pisana Scientific Institute for Research, Hospitalization and Health Care, Rome, Italy

**Keywords:** Functional cure of HIV/AIDS, Viral reservoirs, Eradication research, Antireservoir therapy, Auranofin, Buthionine sulfoximine, SIVmac251 infection, Macaque AIDS model

## Abstract

**Background:**

HIV infection persists despite antiretroviral treatment (ART) and is reignited as soon as therapies are suspended. This vicious cycle is fueled by the persistence of viral reservoirs that are invulnerable to standard ART protocols, and thus therapeutic agents able to target these reservoirs are needed. One such agent, auranofin, has recently been shown to decrease the memory T-cell reservoir in chronically SIVmac251-infected macaques. Moreover, auranofin could synergize with a fully suppressive ART protocol and induce a drug-free post-therapy containment of viremia.

**Results:**

We administered buthionine sulfoximine (BSO), an inhibitor of glutathione synthesis currently in clinical trials for cancer, in combination with auranofin to chronically SIVmac251-infected macaques under highly-intensified ART (H-iART). The ART/auranofin/BSO therapeutic protocol was followed, after therapy suspension, by a significant decrease of viral RNA and DNA in peripheral blood as compared to pre-therapy levels. Drug-free post-therapy control of the infection was achieved in animals with pre-therapy viral loads ranging from values comparable to average human set points to levels largely higher. This control was dependent on the presence CD8^+^ cells and associated with enhanced levels of cell-mediated immune responses.

**Conclusions:**

The level of post-therapy viral set point reduction achieved in this study is the largest reported so far in chronically SIVmac251-infected macaques and may represent a promising strategy to improve over the current “ART for life” plight.

## Background

Antiretroviral therapy (ART) has dramatically improved the clinical conditions of people living with HIV/AIDS, but, unfortunately, its effects are not sufficient to eliminate the virus from an infected host. Following therapy suspension, the virus promptly rebounds to its original pre-therapy viral set point, thus rendering ART a lifelong necessity [1]. Since complete elimination of the virus from the body is still a distant goal, a credible alternative to current ART regimens could be the induction of a functional cure, *i.e.* a condition “in which the virus is not eliminated but is controlled effectively by antiviral immune responses so that drug treatment can be withdrawn for prolonged periods of time [2,3]”. Thus, in the best-case scenario, “functionally cured” individuals should mirror the ability of a small subset of HIV-infected subjects (*i.e.* élite controllers) to arrest disease progression after acute infection in the absence of therapeutic interventions [4]. It follows that viro-immunological parameters associated with élite control may serve as a useful term of comparison for the evaluation of intermediate therapeutic results aiming at a “functional cure”. In this regard, an animal model recently developed by Pandrea *et al.* allowed studying the viro-immunological dynamics associated with élite control [5].

Apart from peculiar genetic and immunological backgrounds, it has been apparent since the early Nineteen- nineties that an obvious correlate of disease progression is the organism’s total viral burden [6]. Moreover, early mathematical modelling showed, from the beginning, a correlation between the extent of the viral burden and the progression of HIV infection either to an “AIDS regime”, *i.e.*, the loss of immune control of the disease [7], or to a stalemate between the virus and the immune system, *i.e.* an “immune state” reminiscent of a functional cure [7].

However, following the discovery of HIV latency [8], it became evident that cellular factors should also be targeted in order to decrease the total virus burden. The sites for the persistence of latent HIV-1 during ART lie in the presence of long-lived viral reservoirs (mainly the memory CD4^+^ T-cell subpopulations), which harbor silent copies of proviral DNA that cannot be targeted by drugs or the immune system. A significant portion of the proviral DNA burden can be found in two subsets, *i.e.* the *central* and *transitional* memory T-cells (T_CM_ and T_TM_, respectively) [9]. Other cell types, however, have emerged as potential reservoirs of latent HIV. Among these, macrophages play an important role in viral propagation as both tissue reservoirs and “Trojan horses” capable of spreading the virus to the central nervous system (for a review, see: Ref. [10]). Candidate anti-reservoir strategies, targeting one or many of these viral reservoirs, may thus exert a profound impact on the viral set point once ART is suspended [11].

In this regard, we recently showed the potent effects in chronically SIVmac251 infected macaques of combined therapeutic protocols targeting both viral replication and cellular factors [12,13]. Such drug combinations, employing antiretroviral drugs and the “anti-memory” compound auranofin, proved able to induce a reduction of the viral reservoir [12,13] and a decrease in the post-therapy viral load set point [12]. These *in-vivo* findings are grounded on *in-vitro* experiments revealing the pro-apoptotic and pro-differentiating effect exerted by auranofin on T_CM_ cells [13] through induction of oxidative stress [14]. In the present study, after further elucidating the viro-immunological effects of auranofin in combination with a highly intensified ART regimen (H-iART), we decided to enhance the *in-vivo* effects of this therapeutic protocol. To achieve this goal, we used buthionine sulfoximine (BSO), a drug that inhibits the synthesis of glutathione, an intracellular antioxidant agent that was previously shown to induce partially selective killing of infected cells *in vitro*[15]. This drug, as well as auranofin, has the relevant advantage of being easily translatable to clinical trials (BSO has been shown to be well tolerated *in vivo* in humans [16], while auranofin has long been employed for treatment of rheumatoid arthritis [17]).

We here show that treatment with auranofin and BSO, in combination with antiretrovirals, results in long-lasting drug-free control of viremia following therapy suspension. This control is dependent on the presence of CD8^+^ cells and is accompanied by an increase in specific immune responses.

## Results

### Combined auranofin/H-iART treatment induces a profound rearrangement in the CD4^+^ T-cell subpopulations

We first analyzed the combined effects of auranofin and a five-antiretroviral drug regimen, *i.e.* H-iART, on the T-lymphocyte subpopulation dynamics and on the immunological post-therapy control of viremia. The initial short-term post-therapy viral set points of these macaques have already been published in Ref. [12].

**Figure 1:**
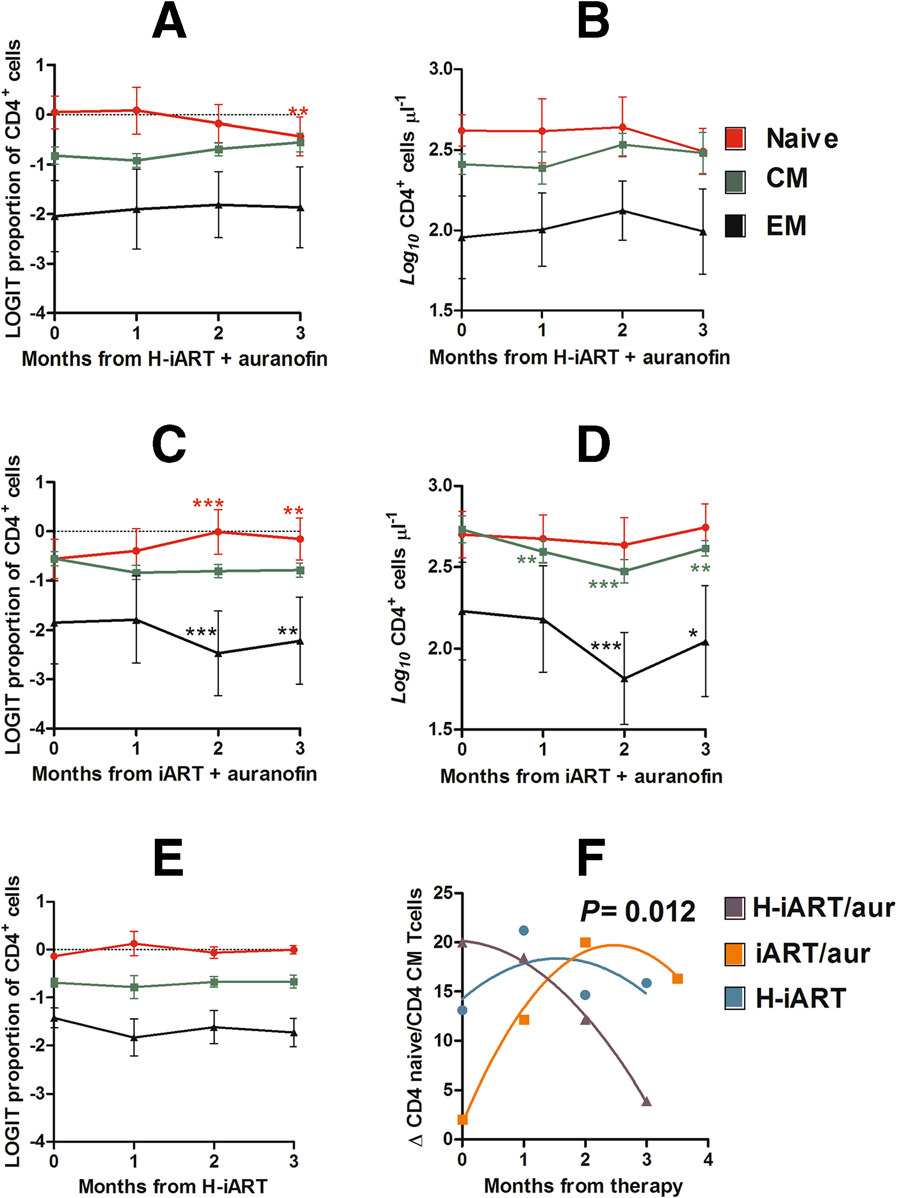
**Combined H-iART/auranofin treatment induces peculiar CD4^+^ T-cell subpopulation rearrangements in SIVmac251-infected macaques.** Panels **A**-**E**. Dynamics over time of percentages (mean and SEM) and absolute numbers (means and SEM) of T_N_ (red), T_CM_ (green) and T_EM_ (black) CD4^+^ T-cells in macaques treated with: auranofin and H-iART (Panels **A**,**B**); auranofin and iART, *i.e.* H-iART without maraviroc (panels **C**-**D**); H-iART only (panel **E**). Total cell counts of macaques treated with H-iART only have been presented in Ref. [12]. Panel **F**. Dynamics over time of the difference between the average relative proportions of T_N_ and T_CM_ cells (Δ T_N_ -T_CM_). Asterisks mark the significant differences with baseline (*i.e.*, time 0) values (* *P* < 0.05; ** *P* < 0.01; *** *P* < 0.001), which were calculated by repeated measures ANOVA followed by Student-Newman-Keuls post-test (panels **A**-**E**). Note that percentage values (panels **A**, **C** and **E**) and absolute values (panels **B** and **D**) were subjected to *LOGIT* or *Log* transformation, respectively, before being subjected to the statistical test. The *P* value in panel **F** refers to the significant difference between curves (extra sum-of-squares *F* test) and was calculated after restoring normality with a *LOGIT* transformation of the data.

CD4^+^ T-lymphocytes of four macaques under auranofin/H-iART were analyzed by flow cytometry. The combined auranofin/H-iART therapy induced a significant reduction in the percentages of naïve cells (T_N_; Figure 1A) and a parallel reduction in the T_N_ absolute numbers, although the latter did not reach statistical significance (Figure 1B).

One of the drugs in the H-iART combination, *i.e.* maraviroc, was previously shown to exert a moderate anti-reservoir effect [12,18] by decreasing antigen-driven proliferation of CD4^+^ T_CM_ lymphocytes [12]. We thus decided to compare the CD4^+^ T-cell subset dynamics under H-iART/auranofin to those of six historical controls [13] treated with auranofin and iART (*i.e.* H-iART without maraviroc). When compared to animals treated with H-iART/auranofin, iART/auranofin-treated macaques displayed a significant increase in the T_N_ percentages (Figure 1C). This increase was paralleled by a decreasing trend in the T_CM_ proportions and by a significant restriction of the T_EM_ percentages (Figure 1C). Absolute cell counts displayed a significant decrease in the T_CM_ and T_EM_ cell subsets (Figure 1D), in accordance with the “anti-memory” effect displayed by auranofin *in vitro*[13].

In order to gain further insight into the role of auranofin in the CD4^+^ T-cell subset dynamics of H-iART/auranofin-treated macaques, we employed a group of three historical controls that had received a cycle of H-iART alone [12]. No definite trend over time was displayed in percentages of any of the subsets (Figure 1E) although absolute cell numbers of T_CM_ and, to a lesser extent T_EM_, displayed a decreasing trend during treatment with H-iART (see Figure six in Ref. [12]).

We conclude that, although H-iART and auranofin may independently decrease the memory T-cell subsets, the combined administration of the two drugs induces more profound rearrangements in the immune system, affecting the T_N_ compartment. This is highlighted by observing the diverging dynamics of a combined parameter including T_N_ and T_CM_ cells (*i.e.* Δ T_N_-T_CM_) in H-iART/auranofin-treated macaques as compared to the other treatment groups (Figure 1F).

Consistent with the subsequent early control of viral load shown by H-iART/auranofin-treated macaques after therapy suspension (see next paragraph), the T-cell subset rearrangements of these macaques are in line with similar T-cell subset dynamics shown in human élite controllers displaying depletion of the T_N_ pools, which continuously renovate the memory subsets subjected to increased turnover [19].

### The combination of auranofin and H-iART changes the pattern of viral rebound and reduces the viral set point following therapy suspension

**Figure 2:**
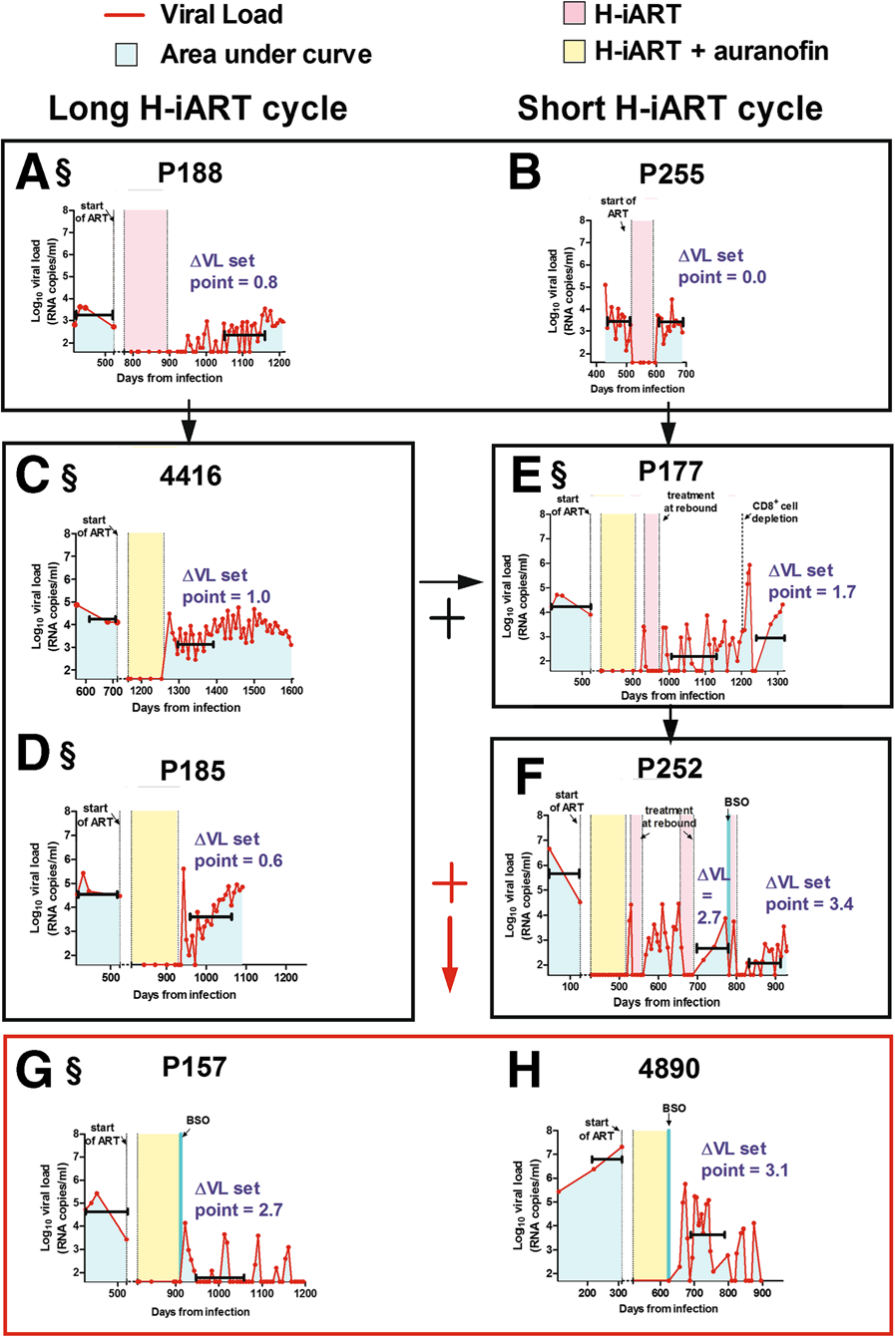
**Evolution of our treatments of SIVmac251-infected macaques: effects on the viral load (VL) set points following treatment interruption.** The figure shows the viral load dynamics of macaques treated with: H-iART alone (Panels **A**,**B**), with either a long (Panel **A**) or a short (Panel **B**) cycle; a combined H-iART/auranofin protocol (Panels **C**,**D**); H-iART/auranofin and a short H-iART cycle at rebound (Panel **E**); H-iART/auranofin and short H-iART cycles at rebound and BSO (Panel **F**); a single cycle of H-iART/auranofin/BSO (Panels **G**,**H**). See main text for further details about the treatment evolution rationale. Horizontal quotes (in black) mark the viral load set points (*y* axes) and the time frames on which their calculations are based (100 ± 15 days; *x* axes). The areas under the viral load curve employed for calculations of the viral set points (see Methods) are depicted in light blue. The symbol ‘§’ indicates the macaques simultaneously treated in the controlled pilot study (see main text for further details). Note that for macaque 4890 (panel **H**) viral loads were measured by NASBA, while real-time PCR was adopted for all the other measurements.

To study the effects of auranofin and H-iART on the post-therapy viral loads, we analyzed the viral dynamics following suspension of H-iART or H-iART/auranofin. Figure 2A-E shows an extended post-therapy time course of the viral loads of macaques treated with H-iART or H-iART and auranofin.

The H-iART treatment was able to induce a reduction, although moderate, in the post-therapy viral set point as compared to pre-therapy levels (Figure 2A,B). This reduction is in line with previous findings suggesting that maraviroc, one of the drugs composing H-iART, is able to impact on the viral reservoir [12,18,20]. In the animals that had received H-iART and auranofin (Figure 2C,D), the decrease in viral set point was higher (0.8 ± 0.2 *Log*_*10*_ viral RNA copies/mL; mean ± S.D) than that of macaques that had received H-iART alone (0.4 ± 0.6 *Log*_*10*_ viral RNA copies/mL). The difference, however, was not statistically significant. In accordance with our previous *in-vivo* experiments with auranofin [13], the addition of auranofin to H-iART changed the pattern of post-therapy viral rebound. The combination induced a peak reminiscent of an acute infection-like condition, followed by a decreased viral set point. Similar to treatments administered during acute infection in humans and macaques [3,21], additional H-iART cycles during the viral load peak at rebound induced a further containment of viremia following therapy suspension as detailed in our previous study (see Ref. [12] and Figure 2E,F).

An experiment conducted in macaque P177 confirmed our previous hypothesis that auranofin treatment followed by H-iART at rebound tilts the virus/immune system balance in favor of the latter [12], since a temporary depletion of CD8^+^ cells at the end of follow-up induced a peak in viral load (up to 5.9 *Log*_*10*_ viral RNA copies/mL, Figure 2E). Measurements of the post-depletion viral loads of macaque P177 showed that, following reconstitution of CD8^+^ cells, the virus was again controlled, though at a level lower than that observed before the depletion (Figure 2E). Whether due to the incomplete CD8^+^ cell reconstitution that occurred following CD8^+^ cell depletion (post-depletion = 445.0 ± 41.8 cells/μL; pre-depletion = 707.6 ± 79.9 cells/μL; *P* < 0.01; Student’s *t*-test), or due to loss of therapeutically induced antiviral responses in the CD8^+^ cell compartment, the poorer post-depletion control of viral load strengthens our published view [12,13] that auranofin induces active immunologic control of viremia and that its effects are not limited to a simple reduction of the reservoir.

### The addition of BSO to H-iART/auranofin induces a drug free containment of viremia following therapy suspension

As the auranofin/H-iART regimen was mainly aimed at targeting the memory T-cell compartment, we decided to potentiate our therapeutic strategy and enhance the pro-oxidant effects of auranofin by adding the glutathione-depleting agent buthionine sulfoximine (BSO) [15]. The rationale for the *in-vivo* use of this drug is supported by a previous study showing the ability of BSO to selectively kill productively infected cells *in vitro*[15], and by new *in-vitro* experiments showing that the combination of BSO and auranofin can induce a “shock and kill” effect on latently HIV-1-infected macrophages (see Additional file 1) which play a major role in the persistence of HIV/SIV *in vivo*[10,22].

We first administered BSO to macaque P252, which had started antiretroviral treatment before the other study macaques. BSO was administered after the auranofin treatment and during an H-iART cycle in temporal vicinity of one rebound in viral load (Figure 2F). The rationale behind this experiment was that the viral rebound induces immune activation which in turn prompts viral escape from latency and consequently renders the infected cells vulnerable to BSO (for a detailed description of the effects of BSO on cells escaping latency see Ref. [15]). Moreover, this experiment allowed an initial evaluation of the effects of BSO in temporal distance from the previous auranofin administration for safety reasons. As a result, we observed an improvement in the drug-free control of viral load following therapy suspension (Figure 2F).

Since the treatment was well tolerated, we devised a simplified therapeutic protocol in which BSO was simultaneously administered to both H-iART and auranofin in macaque P157. This treatment was performed concomitantly to the aforementioned H-iART-only treatment of macaque P188 and the aforementioned H-iART/auranofin-only treatments of macaques P185, 4416 and P177. Following suspension of H-iART/auranofin/BSO, we observed a remarkable 2.7-*Log* reduction in the viral set point (Figure 2G), much higher than that observed in the other study subjects (Figure 2A-F).

We then replicated the combined H-iART/auranofin/BSO therapeutic intervention in another macaque (4890), using a different viral RNA detecting technique (*i.e.* NASBA, see Ref. [23]) to exclude any biases in viral load detection. The H-iART/auranofin/BSO therapy induced a drastic reduction in the post-therapy viral set point (3.1 *Log*_*10*_ viral RNA copies/mL at 100 days post-treatment, Figure 2H), similar to that observed in macaque P157 (Figure 2G). Moreover, the drug-free control of viral load remarkably improved over time (Figure 2H). Of note, this result was obtained in an animal starting from a viral load of 7.3 *Log*_*10*_ RNA copies/mL (*i.e.* 10 fold higher than viral loads typically observed in humans with full-blown AIDS).

This result was subjected to an extensive validation to ensure that post-treatment viral loads obtained in the chronic phase of the disease did reflect an actual *in vivo* phenomenon and were not due to limitations of the technique. In order to rule out false negative results in 4890, we first analyzed the relationship between low (<1000 copies of viral RNA/mL) or undetectable viral loads and the capacity of the assay to detect a viral RNA input run in parallel. When plasma samples of 4890 showed an undetectable viral load, the RNA input was always properly detected and the quantification of its RNA levels yielded results that were comparable to the quantity of input RNA employed, thus proving that the low or undetectable viral loads measured in the samples were not due to technical errors (Additional file 2). Conversely, all our negative controls resulted in no amplification when viral loads peaked, supporting the idea that the viral blips were due to the periodically rebounding virus and not due to contamination within the assay. Moreover, the results of our assay are supported by the viro-immunological correlates, with the major peaks in viral load being accompanied by decreases in CD4 counts, and viral load containment being paralleled by increases in CD4 counts, as is shown below.

**Figure 3:**
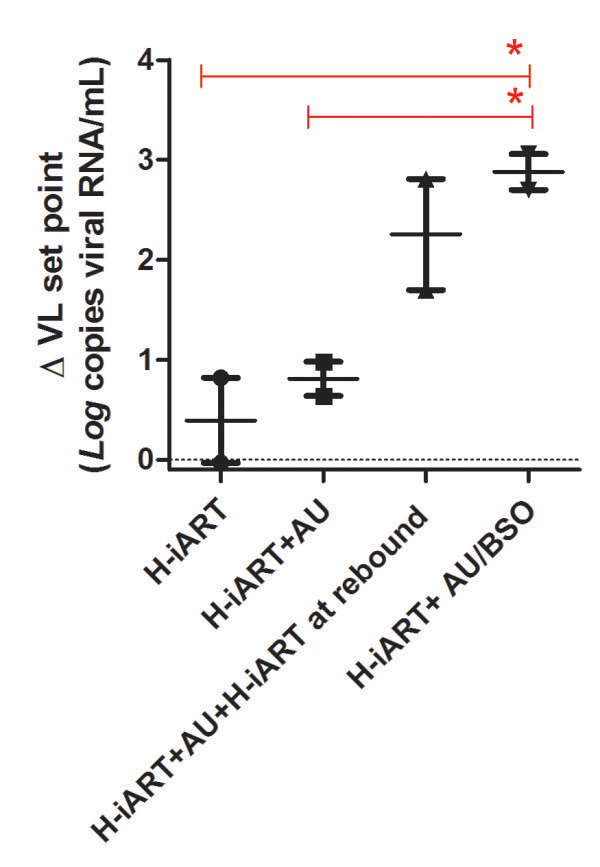
**Comparison of the effects of different therapeutic regimens on the decrease in post-therapy viral set point.** The figure compares the Δ viral load (VL) set point (*i.e.* the difference between the pre-therapy and the post-therapy viral load set points) of macaques subjected to the different therapeutic interventions described in the manuscript. Data have been analyzed by One Way ANOVA followed by Newman-Keuls post-test. Of note, macaque P252, despite being treated with BSO at a later stage, has been included in the treatment group H-iART + auranofin followed by a cycle of H-iART at viral rebound to allow performing the statistical test. However, for this analysis, only the viral set point before treatment with BSO has been taken into account. Significant differences are shown by the horizontal quotes.

Finally, that the abatement of the viral set point was associated with the auranofin/BSO treatment is statistically supported by ANOVA analysis (Figure 3). The effect of H-iART/auranofin/BSO is also supported by other statistical tests such as multivariate analysis and partial correlation analysis, both showing that the number of drugs simultaneously administered to the macaques was the only independent predictor of the lowering of the viral set point out of a group of potential predictors (multivariate analysis: *P* = 0.012; partial correlation: *P* = 0.010). Multivariate analysis also reported a trend between the difference of pre- and post-therapy viral set points and the total number of therapeutic cycles, although this trend did not reach statistical significance (*P* = 0.095).

### Viral load control following suspension of H-iART/auranofin/BSO is associated with maintenance of decreased viral DNA levels in peripheral blood

**Figure 4:**
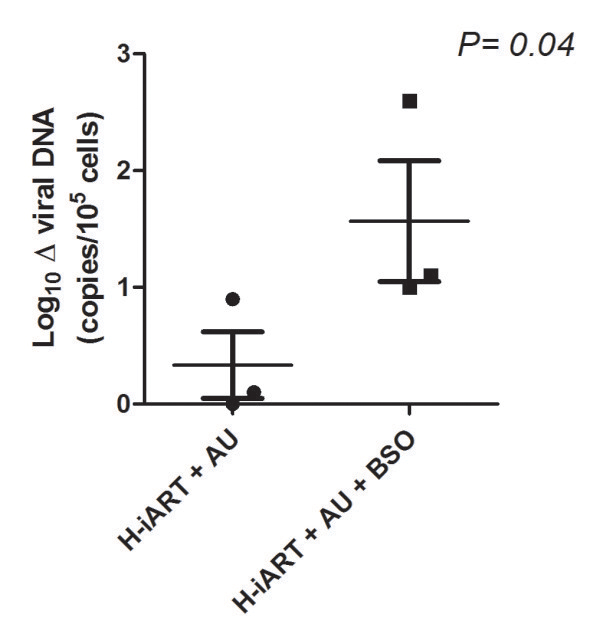
**Comparison of post-therapy viral DNA reduction in animals treated with H-iART/auranofin (AU) and H-iART/auranofin/BSO.** Delta (Δ) viral DNA is shown as the *Log*_*10*_ difference between the medians of pre-therapy and post-therapy viral DNA levels (limit of detection of the assay: 2 DNA copies/5*10^5^ cells). Means and SEM are displayed in the figure. Pre-therapy values represent those at start of the H-iART therapy. To avoid biases (*i.e.* false negative values), post-therapy values were calculated from all available measurements (time window: 189 ± 55 days) following the novel acute infection phase at viral rebound. Data were analyzed by two tailed Student *t*-test. Macaques were matched by similarity of treatments and pre-therapy viral load set point.

To broaden our knowledge of viral containment following therapy suspension, we analyzed the levels of viral DNA in PBMCs of six macaques treated with H-iART/auranofin, with or without BSO. Given the high potency of the antiretroviral therapy employed in this study, it was not possible to directly analyze the contribution of auranofin to the decrease in viral DNA during therapy, which had been demonstrated in a previous study [13]. This impossibility was derived from the fact that H-iART alone, before the addition of auranofin, had *per se* been able to decrease viral DNA in PBMC below the sensitivity threshold of our assay, *i.e.* two copies per 5*10^5^ PBMCs [12]. It was, however, possible to estimate the contribution of BSO to the post-therapy viral DNA levels (Figure 4). All animals (n = 3) that had received H-iART/auranofin/BSO displayed a reduction in post-therapy viral DNA levels in PBMCs, as compared to pre-therapy levels (mean reduction: 1.6 *Log*_*10*_ DNA copies, Figure 4). Instead, only two out of three animals treated with H-iART/auranofin displayed a reduction in viral DNA, and the extent of this reduction was significantly lower than that observed in the BSO-receiving group (*i.e.* 0.5 *Log*_*10*_ DNA copies, Figure 4). We conclude that the H-iART/auranofin/BSO drug combination decreases viral DNA in accordance with the improved control of viremia following therapy suspension.

### Treatment with H-iART/auranofin/BSO results in different CD4^+^ post-therapy equilibriums following therapy suspension

**Figure 5:**
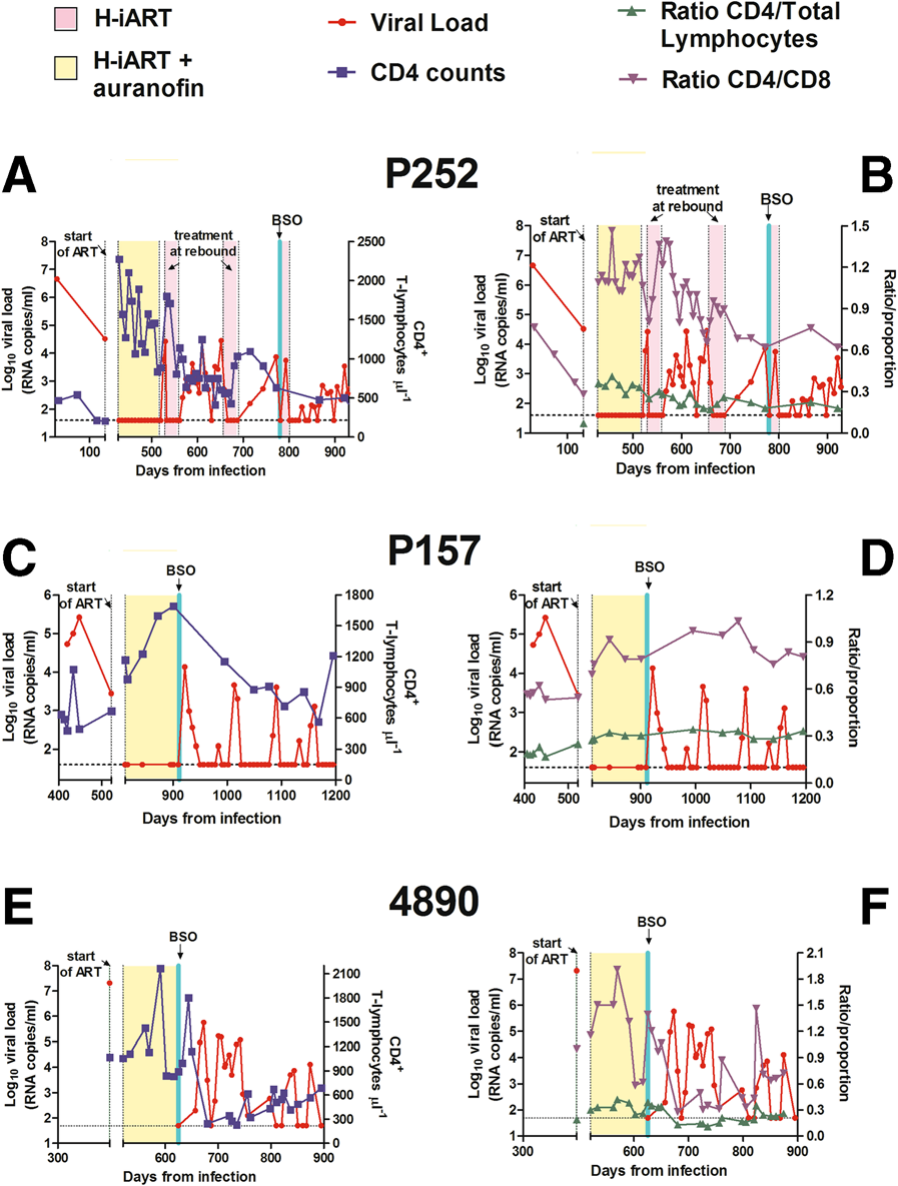
**Viro-immunological parameters of macaques treated with H-iART/auranofin and BSO.** Depicted are absolute CD4^+^ cell counts (blue), proportion of CD4^+^ T-cells over total lymphocytes (green), CD4/CD8 ratio (violet), and viral loads (red). Panels **A**,**C**,**E**: Temporal relationship between viral load and absolute CD4 counts. Panels **B**,**D**,**F**: Temporal relationship bewtween viral load, proportion of CD4+ T-cells and CD4/CD8 ratio. The proportion of CD4^+^ T-cells is shown in a scale between 0 (*i.e.* 0%) and 1 (*i.e.* 100%).

We investigated the viro-immunological correlates of the three animals that had received H-iART/auranofin and BSO. To this aim, we measured the total CD4^+^ cell numbers, the proportion of CD4^+^ T-lymphocytes over total lymphocytes and the CD4/CD8 ratio, before, during, and after therapy (Figure 5). In the animal to which BSO had been administered simultaneously with an H-iART cycle following viral rebound (macaque P252), CD4^+^ T-cell counts gradually returned to a level higher than those prior to therapy but lower than those during therapy (Figure 5A). The same trend was evidenced by analyzing the proportion of CD4^+^ T-cells over total lymphocytes and the CD4/CD8 ratio (Figure 5B). One of the animals that had received a single H-iART/auranofin/BSO therapeutic cycle (macaque P157) also displayed the same trend in CD4^+^ T-cells over time (Figure 5C-D), but maintained higher CD4 counts (898 ± 227 cells/μL, mean ± SD; Figure 5C), which were associated with a higher proportion of CD4^+^ T-cells and a normal CD4/CD8 ratio (Figure 5D). This animal was subjected to an extended follow-up which proved that, one year after therapy interruption, along with an undetectable viral load, total CD4^+^ T-cell counts were still high (*i.e.* 1210 cells/μL, Figure 5C). The viro-immunological parameters of this macaque following therapy suspension met the definition of “functional cure” previously adopted in this Journal [3].

An animal model for a functional cure of AIDS was recently obtained by Pandrea *et al.*[5] by infecting non-natural hosts (Rhesus macaques) with a primate lentivirus derived from African green monkeys (SIVagm). In this model, following an initial viral load peak accompanied by CD4^+^ T-cell depletion, viral loads were eventually controlled to undetectable levels for prolonged periods of time, and CD4 counts gradually increased over time. To investigate whether there might be any similarities between the model described by Pandrea *et al.* and the acute infection-like viral load peak followed by improved containment of viral load in our macaques, we decided to monitor the viro-immunological correlates of the early phases of viral rebound following suspension of H-iART/auranofin/BSO. Although it was not possible to monitor the early post-therapy phases of the CD4^+^ T-cell dynamics in macaque P157, these phases could be followed-up in the second animal (macaque 4890) that had received the single H-iART/auranofin/BSO therapeutic cycle (Figure 5E,F). Although this macaque promptly controlled a major viral rebound following therapy suspension, it initially displayed a partial CD4^+^ T-cell depletion (Figure 5E). The proportions, and absolute numbers of CD4^+^ T-cells, as well as the CD4/CD8 ratio, however, gradually increased over time (Figure 5F).

**Figure 6:**
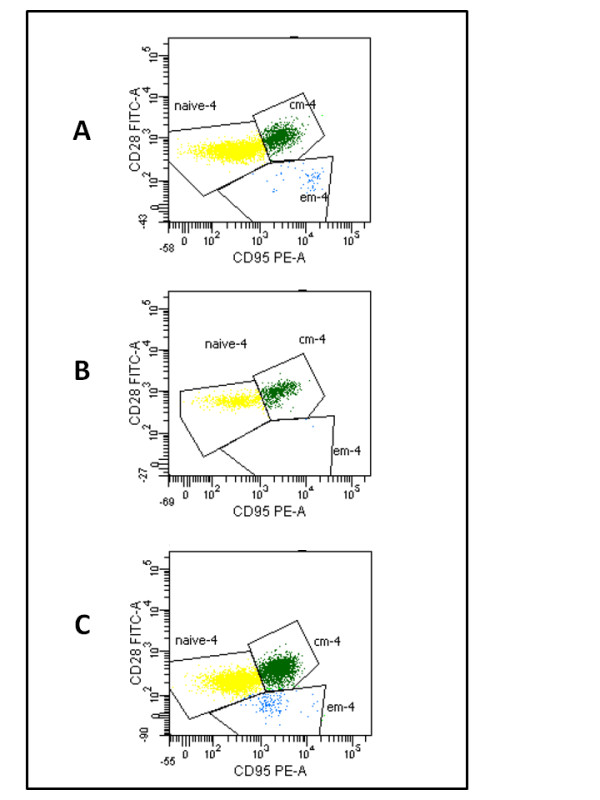
**Dynamics over time of CD4^+^ T-cell subsets in macaque 4890.** Shown are the naïve CD4^+^ T-cells [naïve-4 (CD28^+^ CD95^-^), in yellow], the central memory CD4^+^ T-cells [cm-4 (CD28^+^CD95^+^), in green] and the effector memory CD4^+^ T-cells [em-4 (CD28^-^CD95^+^), in blue] of macaque 4890, as measured on three occasions: under therapy (panel **A**), three months post-therapy interruption (panel **B**), eight months post therapy interruption (panel **C**).

As, in the model of Pandrea *et al.,* the CD4^+^ T_EM_ subset was the most depleted cell subpopulation during the acute SIVagm infection, we investigated the dynamics of this T-cell population in macaque 4890. Flow cytometrical analysis of circulating CD4^+^T-cells, showed that the CD4^+^ T_EM_ cell subset was initially depleted following viral rebound and was reconstituted in parallel to improvement of viral load control (Figure 6). In conclusion animal 4890 showed an initial acute infection-like condition after therapy suspension that was followed by improved control of viral load and an increasing trend toward normalization of the immunological parameters.

The H-iART/auranofin/BSO-treated macaques P157 and 4890 were still alive at the time when the present manuscript was written, and had not shown any AIDS-related events during the follow-up.

### Post-therapy control after treatment with H-iART/auranofin/BSO is likely to be associated with enhancement of specific immunity

Previous studies have shown the role of specific immunity in viral load containment both during the acute and chronic phases of the disease [24,25]. We thus analyzed whether the post-therapy control of viremia following treatment with H-iART/auranofin/BSO was associated with changes in specific immune responses. We first tested the effects of our treatments on the levels of neutralizing antibody titers, assessed in the TZM-bl assay as described in Ref. [26]. We found that BSO-containing therapies transiently increased neutralizing antibody titers in only one of three animals (Additional file 3). No significant changes (>2.5 fold) were detected in a control animal treated without BSO (Additional file 3).

**Figure 7:**
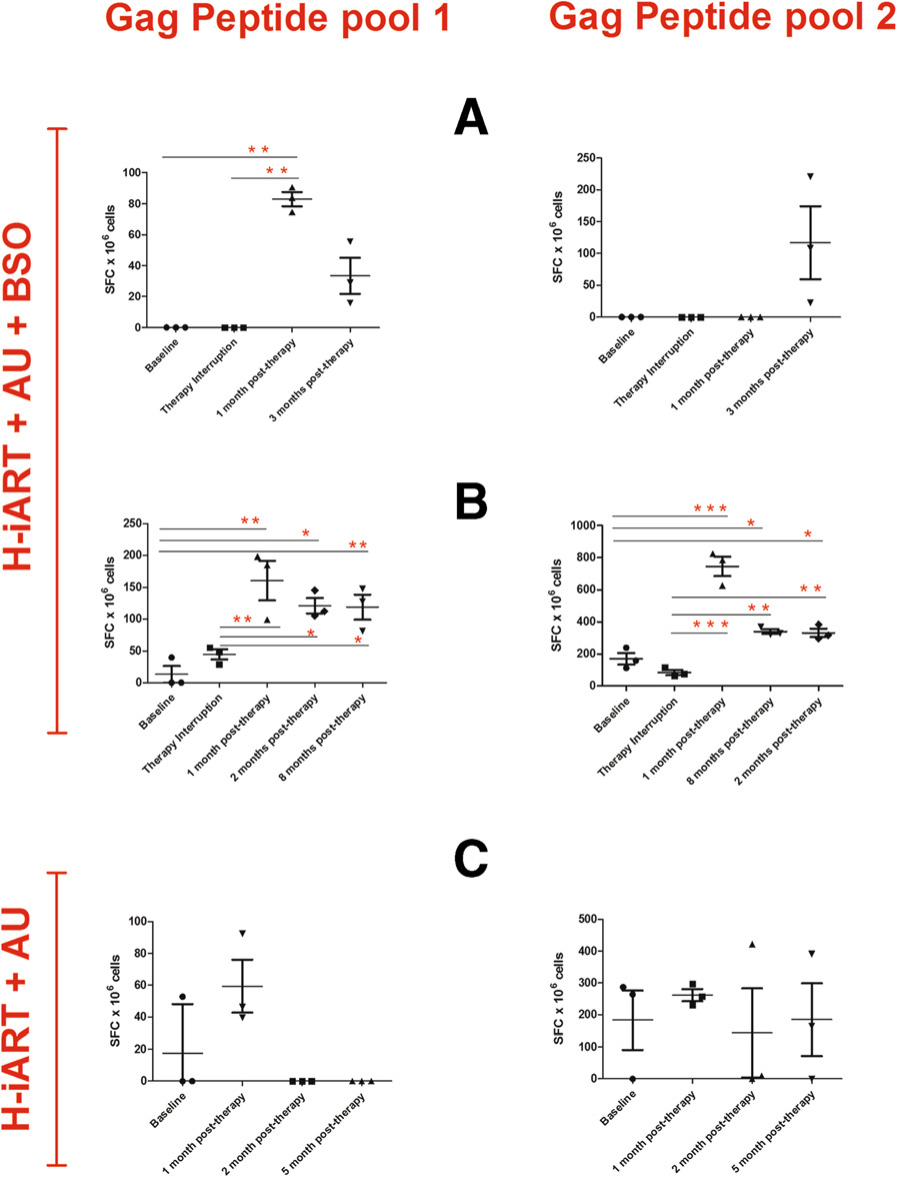
**ELISpot detection of gag specific immune responses of SIVmac251 infected macaques treated with H-iART/auranofin(AU)/BSO or H-iART/auranofin.** PBMCs were stimulated with two separate SIV Gag peptide pools spanning the entire Gag protein sequence. Data are presented as the number of spot forming cells (SFC) per million PBMCs. Panels A,B show the results from macaques treated with H-iART/auranofin/BSO (Panel **A**: macaque 4890 and Panel **B**: macaque P157). Panel **C** shows, as a matter of comparison, the anti-SIV cell-mediated responses of a macaque (P185) that had been treated with H-iART/auranofin without BSO. Numbers of SFC per well were normalized per 10^6^ cells after subtracting the average background (media only) SFC per well. Data were analyzed by repeated-measures ANOVA, followed by the Student-Newman-Keuls post-test. Asterisks mark the statistically significant differences between the baseline levels (during therapy) or those at therapy interruption) and the post-therapy time points: * *P* < 0.05; ** *P* < 0.01; *** *P* < 0.001.

**Figure 8:**
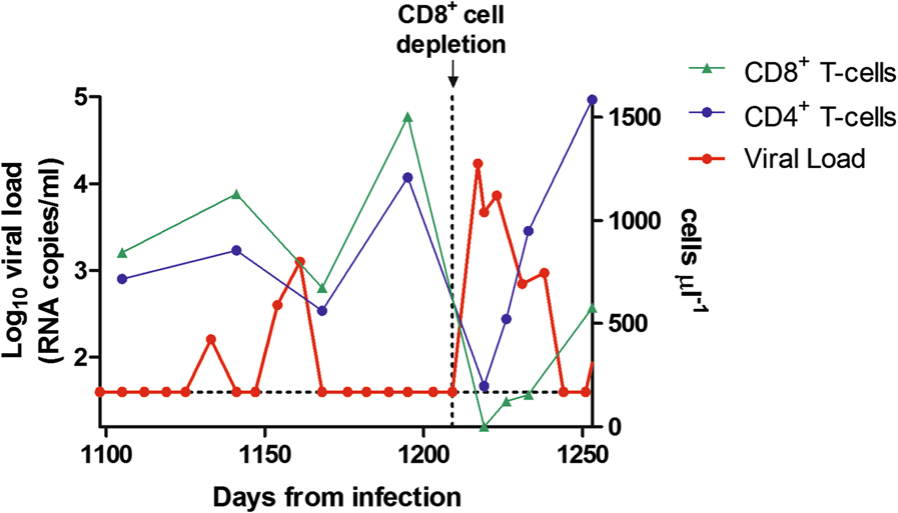
**Long term post-therapy viral load control following H-iART/auranofin/BSO treatment is dependent on CD8^+^ cells.** CD8^+^-cell depletion was induced in macaque P157 using the anti-CD8 monoclonal antibody cm-T807. The dotted line parallel to the *x*-axis represents the limit of detection for measurement of viral load (*i.e.* 40 viral RNA copies/mL of plasma).

We also monitored cellular immune responses to the Gag protein in animals that had received the combined H-iART/auranofin/BSO protocol. These animals displayed a significant increase in SIV Gag-specific ELISpot responses following treatment interruption, as compared to animals that had received H-iART/auranofin without BSO (Figure 7A-C). This increase was sustained for at least eight months (Figure 7B). Of note, these animals did not display any of the protective MHC allele combinations (see Table S1 of Ref. [12]). To further elucidate the contribution of CD8^+^ cells to the preservation of post-therapy viral load control, we induced CD8^+^-cell depletion by means of a monoclonal α-CD8 antibody [25] in one of the macaques that had received H-iART/auranofin/BSO (macaque P157). The depletion of CD8^+^ cells was accompanied by a peak in viral load (up to 4.2 *Log*_*10*_ viral RNA copies/mL of plasma), while CD8^+^ T-cell reconstitution was paralleled by a return to the previous viral load steady state (Figure 8).

The viral rebound induced by CD8^+^-cell depletion was accompanied by a transient CD4^+^ T-cell depletion (Figure 8), proving that the virus, although kept at low or undetectable levels following therapy suspension, was replication-competent and pathogenic. Moreover, a viral cultivation experiment using a mixed cell population of pre-depletion PBMCs and uninfected CEMx174 cells showed that the virus was both detectable with a p27 ELISA and cytopathogenic (data not shown). We conclude that post-therapy control of viremia after suspension of H-iART/auranofin/BSO was not due to lack of replicative capacity or infectivity of the virus, and that CD8^+^ cell-mediated responses might play a crucial role to keep the virus in check.

## Discussion

### Likely advantages of H-iART/auranofin/BSO over previous regimens

In the present study, we observed a drug-free control of viremia following suspension of therapies containing antiretroviral drugs and agents targeting cellular factors. The contribution of the glutathione depleting agent BSO to post-therapy viral load containment was first tested in a small controlled pilot study in which the animals were simultaneously treated with therapeutic regimens consisting of H-iART alone (n = 2) or a combination of H-iART and auranofin, with (n = 1) or without (n = 3) BSO. The addition of BSO was associated to an apparent improvement in post-therapy control of viremia that resulted in a functional cure-like condition of the treated animal. This observation is in line with observations deriving from two additional animals that had received BSO and had displayed a reduction in the post-therapy viral set point of >3 Log_10_ copies of viral RNA per mL of plasma. We previously showed that one similar control can be induced by preventing viral reservoir re-expansion through a potent antiretroviral (H-iART) cycle at virus rebound [12]. The current BSO-based approach, instead, counteracted the rebounding virus likely by reinforcing the immune responses, as suggested by ELISpot analyses and shown by the prompt virus rebound that followed CD8^+^ cell depletion. However, it should be noted that the ELISpot assay, despite being a standard tool for detection of specific immune responses, has the typical limitations common to antigen driven assays, and further analyses will be necessary for in-depth investigation into the immune control of the post-therapy viral rebound.

It is as yet unclear whether the boost of the immune responses is due to auranofin and BSO specifically, or is a consequence of suppression and rebound of virus in a partially immune reconstituted host.

On one hand, immune enhancement by two oxidative stress-modulators such as auranofin and BSO [27-30] is not surprising in light of the observation that reactive oxygen species (ROS; *e.g.* H_2_O_2_ and O^2-^) are mitogenic via changes in protein phosphorylation and/or activation/inhibition of transcription factors [31]. For example, the combined use of a pro-oxidant agent (auranofin) and a GSH depleting one (BSO) may enhance intracellular H_2_O_2_ production, in turn favoring the early events of lymphocyte activation by inducing NF-κB as well as IL-2 and IL-2 receptor α-chain gene transcription [32]. Moreover, oxidative stress has been previously shown to enhance antigen immunogenicity by facilitating antigen presentation to both MHC I and II [33-36].

On the other hand, the viral rebound following therapy suspension may have acted as an “endogenous” vaccine able to induce a robust immune response against the virus. Of note, durable (*i.e.* >9 months) control of viral load was observed only in those macaques that had received both auranofin and BSO.

However, the macaques treated with H-iART/auranofin/BSO were selected, on purpose, among those starting with a higher pre-therapy viral load set point in order to highlight the robustness of the results obtained with this therapy. This higher pre-therapy viral load set point may have resulted in an increased antigenic stimulation which, coupled with the reduction of the viral burden and the partial immune reconstitution observed during therapy, may have contributed to the enhanced immune responses associated with the drug-free control of viremia.

### Association between drug-free containment of viremia and effects on virus-target cells

The enhanced immune response following treatment with auranofin/BSO is likely to be associated with restriction of the circulating pools of viral DNA following therapy suspension. We previously published results consistent with a model wherein the elimination of the memory cell pools (which are a major target for viral replication, encompass one major viral reservoir and are exhausted by SIV infection) would have a favorable impact on post-therapy disease progression [13]. Although, in the present study, the memory T-cell compartment was targeted by both auranofin and maraviroc, we found that the H-iART/auranofin regimen mainly affected the CD4^+^ T_N_ percentages (Figure 1). This apparent discrepancy may be reconciled by the view that targeting the memory T-cell compartment by a double mechanism could spark a compensatory differentiation of T_N_ into T_CM_ and T_EM_ lymphocytes. That an increased T-cell turnover results in decreased T_N_ is in line with the restricted T_N_ pools observed in élite controllers, whose memory T-cells are subjected to increased turnover due to maintenance of viral replication under check [19]. While this increased lymphocyte turnover could contribute to both immune renovation and elimination of the infected cells, a further rise in this turnover following therapy suspension could induce, as a “byproduct”, a reduction in the T-cell regenerative capacity. These considerations may explain the maintenance of relatively low T-cell counts in one of the macaques that had received H-iART/auranofin/BSO, despite the large decrease in the post-therapy viral set point. Our results are in line with the low CD4^+^ T-cell counts displayed by some nonhuman-primate and human élite controllers [5,19]. Thus, maintenance of CD4 counts to relatively low levels, but consistent with an unimpaired specific immunity, may represent a price paid by the host for keeping viremia under check. Understanding whether the lowered CD4 counts may provide a target restriction effect [37] will require further investigation.

Part of the rationale behind our therapeutic strategy is based on the “shock and kill” effect exerted *in vitro* by the combination of auranofin and BSO on infected macrophages (Additional file 1). As these cells are rare, studies using larger number of macaques will be necessary in order to allow drawing statistically accurate conclusions on the effects of our drug combination on this viral sanctuary *in vivo*.

### Similarities and differences with other conditions of drug-free remission of AIDS

Elimination/restriction of the virus target cells plus immune restoration represents a promising approach that has also been followed in order to obtain the first cure of AIDS in Mr. Timothy Brown, the “Berlin patient” [38]. In that case, CD4^+^ T-cells were eliminated by total immune ablation and immune system renovation was guaranteed by stem cell transplantation. However, the replacement of Mr. Brown’s HIV target cells with cells that the virus cannot infect renders a comparison to our approach difficult.

Recently, a drug-induced functional cure in the macaque AIDS model has been published by Van Rompay *et al.* in this Journal [3]. In the macaques of Van Rompay, formation of the infected cell pools may have been limited by the relatively early treatment and decreased virus replicative capacity, and CD8^+^ cell depletion during therapy may have induced immune system renovation. A striking similarity with the results of the present study can be found in the fact that the macaques of Van Rompay showed, upon therapy suspension, periodical peaks in viral load that were followed by containment to very low or undetectable levels. Similar viral load peaks followed by containment to undetectable levels were also observed in other “functional cures” obtained treating early during acute infection both in SIV-infected macaques and in the “VISCONTI patients” [39-41]. The amplitude of the peaks observed is higher in the “functional cures” of macaques as compared to those described in humans [39,41]. This discrepancy can be explained by considering the viral burst size (*i.e.* the average number of viral particles produced from a single infected cell), which is one order of magnitude higher for SIV than for HIV-1 [42]. A visual example of these phenomena is shown by the mathematical simulations in Additional file 4.

Finally, the viro-immunological correlates of one of our macaques after suspension of treatment with H-iART/auranofin/BSO were reminiscent of the virus/CD4^+^ T-cell dynamics observed in the model of Pandrea *et al.* for a functional cure of AIDS in macaques [5], with CD4^+^ T-cell depletion associated with higher viral loads in an initial phase and CD4^+^ T-cell restoration in a later phase characterized by improved containment of viral load.

## Conclusions

Our data suggest that a combined H-iART/auranofin/BSO treatment was followed by a radical change in disease progression after therapy suspension. This change was associated with enhanced specific immune responses following viral rebound after therapy suspension, thus suggesting the establishment of an “immune state” [7]. Similar effects had previously been observed only in the period immediately after acute infection but not in a pre-AIDS stage [21,39-41]. Although fully controlled studies involving larger numbers of macaques will be necessary in order to obtain further mechanistic insight, it is becoming increasingly evident that dramatic changes in disease progression cannot be evaluated only in terms of sheer numbers of study subjects [38,43]. Whether these results may pave the way to a significant improvement of current “ART-for-life” therapies will need to be assessed in human clinical trials.

## Methods

### Animal treatment

The Indian rhesus macaques used in this study were housed at Bioqual, Inc., according to standards and guidelines as set forth in the Animal Welfare Act, the Guide for the Care and Use of Laboratory Animals, and the Association for the Assessment and Accreditation of Laboratory Animal Care (AAALAC), following approval by the Institutional Animal Care and Use Committee (IACUC). Eight SIVmac251-infected macaques in the chronic phase of the infection were enrolled in this study starting from June 2010. The baseline viro-immunological conditions of the study subjects and their antiretroviral treatments preceding April 2012 have been described in detail in a recent publication [12]. Macaques in the late chronic phase of the infection displaying antiretrovirally suppressed viremia (*i.e.* < 50 copies/mL) were kept under highly-intensified ART (H-iART; consisting of tenofovir, emtricitabine, raltegravir, ritonavir-boosted darunavir and maraviroc) and auranofin for three months. Two macaques were similarly kept under H-iART in the absence of auranofin to serve as controls. Additional H-iART cycles (mean duration: 31 days) were administered to animals P177 and P252 in order to decrease viral reservoir replenishment at viral rebound. Auranofin (Prometheus Laboratories, San Diego, CA) was administered by the oral route twice daily with food (0.4 mg/kg/day). BSO (Sigma-Aldrich, St.Louis, MO, USA) was administered to three macaques intraperitoneally at 450 mg/kg every eight hours for a total of five administrations (the details of the timing and its rationale are given in the Results section). All animals were dosed subcutaneously with tenofovir, and emtricitabine, and orally (with food) with raltegravir, ritonavir-boosted darunavir, and maraviroc. Drug dosages were: tenofovir, 30 mg/kg/day; emtricitabine, 50 mg/kg/day; raltegravir, 100 mg bid; darunavir, 375 mg bid (for macaques starting from viral loads lower than 10^5^ viral RNA copies/mL) or 700 mg bid (for macaques starting from viral loads higher than 10^5^ viral RNA copies/mL); ritonavir 50 mg bid; maraviroc 100 mg bid. Tenofovir and emtricitabine were kindly provided by Gilead Sciences (Foster City, CA). Raltegravir, DRV/r and MRV were purchased from the manufacturers.

Pilot treatments of macaques P255 and P252 were conducted in 2010/early 2011. Treatments of macaques P157, P177, P185, P188, and 4416 were conducted simultaneously in 2011. An additional SIVmac251-infected animal (4890) was treated in 2012 in order to ensure reproducibility of the results.

Macaques P157 and P177 were subjected to CD8^+^ cell depletion after therapy suspension in order to study the contribution of CD8^+^ cells to post-therapy viral load control. The depletion was performed with the previously validated antibody cm-T807 ([25,44] kindly provided by the NIH Nonhuman Primate Reagent Resource center). The antibody was administered on four different occasions (days 0, 3, 7 and 10) at 10 mg/kg subcutaneously for the first administration and at 5 mg/kg intravenously for the three remaining administrations.

### Quantitative assay for SIVmac251 viral RNA levels

For measurement of plasma SIVmac251 RNA levels, a quantitative TaqMan RNA reverse transcription-PCR (RT-PCR) assay (Applied Biosystems, Foster City, CA, USA) was used, which targets a conserved region of the *gag* transcripts. The samples were then amplified according to a method previously described in [12,45]. The sensitivity of the method is two copies per run, which results in a detection limit as low as 40 RNA copies/mL in our routine analyses. This method and its validation data are described extensively in Ref. [12]. For the SIV real-time NASBA assay, macaque plasma was clarified by centrifugation at 2300 × g for 3 mins. The clarified plasma was either lysed directly (0.1 mL) in lysis buffer (bioMerieux, Durham, NC, USA) or further centrifuged to pellet virus from a higher volume (0.5–1 mL) by ultracentrifugation at 49 100 × g for 60 min. The virus pellet was then lysed in 1 mL lysis buffer. A fixed amount of Q calibrator RNA (10^5^ copies or 10^4^ copies) was added to the lysed sample and the nucleic acid was extracted using acidified silica as described previously [46]. The quantitative range of the assay was determined by the concentration of the calibrator added [47]. For samples expected to have a viral load >10^4^ copies/mL, 10^5^ copies of calibrator RNA were used. However, for lower loads, 10^4^ copies of Q calibrator RNA were used.

The SIV real-time amplification final reaction volume was 20 μl, containing 5 μl of nucleic acid extract and 40 mM Tris, pH 8.5; 12 mM MgCl2; 90 mM KCl; 5 mM dithiothriotol; 1 mM each dATP, dCTP, dGTP, dTTP; 2.0 mM each ATP, CTP, UTP; 1.5 mM GTP; 0.5 mM ITP; 0.1 ìg/ìl BSA; 1.5 M sorbitol; 0.08 units RNase H; 32 units T7 RNA polymerase; 6.4 units avian myeloblastosis virus reverse transcriptase (AMV-RT); 0.01 μM SIV WT molecular beacon probe; 0.1 μM SIV Q molecular beacon probe; 0.2 μM each of the two amplification oligonucleotides, and 15% DMSO. The amplification oligonucleotides used are as follows—P1: 5′-AATTCTAATACGACTCACTATAGGGCACCAGATGACGCAGACAGTATTA-3′; P2: 5′-CTCCGTCTTGTCAGGGAAGAAAGCA-3′. The SIV WT molecular beacon has the fluorophore FAM linked to the 5′ end and a quencher linked to the 3′ end; 5′-FAM-CGATGCATGTAGTATGGGCAGCAAATGAAGCATCG-DABCYL-3′. The SIV Q molecular beacon has the fluorophore 6-ROX linked to the 5′ end and a quencher linked to the 3′ end; 5′-6-ROX-CGATGCGTTGAAGTGCAGTAGTGATGGCATCG-DABCYL-3′. All reagents, except for enzymes and BSA, were mixed and preincubated at 65°C for 2 min. The reaction mixture was then cooled for 2 min at 41°C and the enzymes and BSA were added. Samples were mixed and placed in the NucliSENS EasyQ Analyzer (bioMerieux, Durham, NC, USA) and isothermal amplification took place at 41°C for 90 min. The NucliSENS EasyQ Analyzer took measurements throughout the amplification reaction, resulting in two fluorescence recovery curves. An algorithm was then applied that uses the kinetics of fluorescence recovery from WT and Q RNA to calculate the SIV RNA copy number in the plasma samples. The SIV RNA load was expressed as viral RNA copies per ml plasma. The inter-assay coefficient of variation of the technique was 19%, well within the limits of the typical inter-assay variability of SIV RNA detection techniques [*e.g.*[12,48].

### Quantitative assay for SIVmac251 proviral DNA

For proviral DNA detection, DNA was extracted with the phenol-chloroform method. Quantification was performed by amplifying a region of the gag gene by real time PCR in a 7700 Sequence Detection System (Applied Biosystems). Details and validations of the technique are extensively described in [12,45].

### Immunofluorescent staining and flow-cytometric analysis

Hematological analyses were performed by IDEXX (IDEXX Preclinical Research, North Grafton, MA). For calculation of absolute CD4^+^ and CD8^+^ T-cell numbers, whole blood was stained with anti-CD3-fluorescein isothiocyanate (FITC)/anti-CD4-phycoerythrin (PE)/anti-CD8-peridinin chlorophyll α protein (PerCP)/anti-CD28-allophycocyanin (APC), and anti-CD2-FITC/anti-CD20-PE, and red blood cells were lysed using lysing reagent (Beckman Coulter, Inc., Fullerton, CA, USA). Samples were run on a FACSCalibur (BD Biosciences, San Jose, CA, USA).

Staining for naïve (T_N_: CD28^+^CD95^-^), central memory (T_CM_: CD28^+^CD95^+^), and effector memory (T_EM_: CD28^-^CD95^+^) T-cells was performed on PBMCs isolated from total blood as described in [12]. The mAbs used (BD Biosciences) were: anti-CD3 (APC-Cy7), anti-CD4 (Per-CP), anti-CD8 (Pe-Cy7), anti-CD20 (APC), anti-CD28 (FITC) and anti-CD95 (PE). Six-parameter flow-cytometric analysis was performed on a FACS Canto II instrument (BD Biosciences). The absolute numbers of T_N_ (CD95^-^CD28^+^), T_CM_ (CD95^+^CD28^+^) and T_EM_ (CD95^+^CD28^-^) memory CD4^+^ T-cells were deduced from percentage values of parent cells.

### Detection of neutralizing antibodies

Sera from different time points during the study were assayed in the TZM-bl assay system [26] against a neutralization-sensitive (SIVmac251.6) and a neutralization-resistant (SIVmac251.30) pseudotyped virus. Virus pseudotyped with Murine Leukemia Virus Env (SVA-MLV) was included to assess non-SIV-specific neutralizing activity in the sera. Briefly, serial dilutions of sera from the indicated time points were pre-incubated with virus (~150,000 relative light unit equivalents) for 1 hr at 37°C. Following addition of TZM-bl target cells, the cultures were incubated for 48 hours, then lysed and assayed for luciferase activity. Neutralization titers are the sample dilution at which relative luminescence units (RLU) were reduced by 50% compared to RLU in virus control wells after subtraction of background RLU in cell control wells. In several instances, ID_50_ endpoint titers against SIVmac251.6 were not achieved in the dilution series employed in the assays. Thus, an 80% neutralization titer (ID_80_) was calculated to expose the relative potency of the neutralizing antibody response against this virus.

### IFN-γ ELISpot assay

Specific immune responses were detected by measuring gamma interferon (IFN-γ) secretion of macaque PBMCs stimulated with SIVmac239 Gag peptides (125 overlapping 15-mer peptides, obtained through the AIDS Research and Reference Reagent Program, National Institutes of Health [NIH]) in an enzyme-linked immunospot (ELISpot) assay. The peptides were resuspended according to the manufacturer’s instructions and divided in two pools (pool 1: peptides 1–63; pool 2: peptides 64–125). The assay was performed with the ELISpotPRO for monkey interferon-γ kit (Mabtech AB, Nacka Strand, Sweden) according to the manufacturer’s instructions. Briefly, 1.5 × 10^5^ Ficoll isolated macaque PBMCs were added to 96 well plates pre-coated with an anti-human/monkey IFN-γ antibody (MAb GZ-4). Cells were resuspended in RPMI 1640 + 10% FBS with or without 5 μg/mL of each peptide pool, or concanavalin as a positive control. Triplicate wells were employed for each experimental condition. After 48 hours incubation at 37°C with 5% CO_2_, the cells were rinsed from the plates, and a biotinylated anti-human/monkey IFN-γ antibody (MAb 7-B6-1; Mabtech) was added to the wells. The plates were then washed with PBS and incubated with the substrate solution (BCIP/NBT-plus). Spot forming cells (SFC) were counted by using an automated reader (Immunospot Reader, CTL analyzers, LLC, Cleveland, OH). Data were expressed as average numbers of SFC/10^6^ cells after subtracting the average number of background spots detected in the negative controls.

### Statistical analyses and mathematical simulations

The inter-assay coefficient of variation of the SIV real time NASBA technique was calculated in accordance with a standard procedure [*e.g.* see Ref. [48]. Two different control samples (containing ≈ 10^4.7^ and ≈ 10^2.7^ copies of SIV RNA copies/mL, respectively) were tested in three different runs per sample. Two coefficients of variation were calculated from the results of these runs and the overall coefficient of variation was calculated as the mean of the two. Differences between variables were calculated using parametric tests (Student’s *t*-test for comparisons between two groups, and ANOVA followed by the Student-Newman-Keuls post-test for multiple comparisons). An appropriate transformation was applied to restore normality, where necessary. Repeated-measures tests were used when analyzing matched observations. Trends were analyzed by regression analysis, followed by the extra sum-of-squares *F* post-test. Calculations were conducted using the software GraphPad Prism 5.00.288 (GraphPad Software, Inc., San Diego, CA, USA).

The viral set point was calculated using the area-under-curve (AUC) method using the GraphPad (v.5) software. To ensure consistency in the results obtained, we considered the post-therapy viral load values in the same time interval as that of the available pre-therapy values. The time frames analyzed in the different macaques were largely comparable, since the standard deviation of the periods considered accounted for only 15% of the mean value. To ensure accuracy in the analyses, the starting value employed for calculation of the post-therapy vial load set point was selected according to the following criteria:

1) For animals that had received auranofin, and had therefore experienced the previously published acute-infection-like condition [13], we started the follow-up immediately after the acute-infection-like peak in viremia. This choice mimics calculations of the viral set point in the natural course of the infection, discarding the acute infection phase as non-representative of the equilibrium reached thereafter. The initial value adopted for our viral load set point calculations was the minimum in the curve after the initial peak.

2) For animals that had the initial viral load peak abated artificially by H-iART, the period under drugs was not taken into account, and the viral set point calculations were started after therapy withdrawal.

3) For animals that had not received auranofin and for which there was no clear initial peak, follow-up was started when viremia plateaued. This allowed discarding the initial values and providing a sensible comparison with the data from the auranofin-treated animals. We considered that the plateau was reached when viral load reached values above 80% of the asymptote of the one-phase association curve describing the viral rebound and obtained by non-linear regression analysis option embedded in the GraphPad software. Additional file 5 provides an illustrated example of the method adopted.

For each of the cases presented in 1), 2) and 3), the input values were analyzed using the “area under curve” option in the GraphPad software. Peaks accounting for less than 10% of the AUC were automatically discarded by the default option of the software adopted. To allow an overall comparison of the results obtained, results were normalized by dividing by the length of the temporal window.

Multivariate analysis was used to study the contribution of independent predictors to the post-therapy viral load set point and the difference between the pre- and post-therapy viral load set points points (Δ viral set point). The analysis was conducted with the IBM SPSS software (v. 21, Armonk, NY, USA), using a type-I regression parameter, and the post-therapy viral set point and the Δ viral set point as dependent variables. The choice of the potentially predicting parameters was based on literature analysis. Possibly independent predictors were considered to be the number of drugs simultaneously employed, the total duration of therapy (*i.e.* the number of days during which macaques were exposed to drugs), the number of therapeutic cycles and the pre-therapy CD4 nadir [12,49]. A “therapy” was considered to be a sequence of therapeutic cycles wherein the distance between neighboring treatments did not exceed 90 days. Partial correlation analysis was conducted using SPSS, starting from the same parameters, and controlling by the total duration of therapy.

For a discussion of mathematical methods and numerical tools employed in the numerical simulations, see the “Statistical and biomathematical analyses” paragraph in the “Materials and methods” section and Text S1 in Ref. [12]. See also Ref. [50]. Briefly, for our simulations we adjusted for a macaque model the system of ordinary differential equations (system 4) introduced in [50]. In this system, a random step function is used to simulate the periods in which there is activation of resting latently infected CD4^+^ T-cells. The activation function used in the simulations was generated with the RANDLIB package of the Scilab 5.3.3 software (freely available at http://www.scilab.org). This function is shown in the figure of Additional file 6. Further information on mathematical modeling is given in the text of Additional file 6 while the starting data employed for the numerical simulations are shown in Additional file 7.

## Abbreviations

ART: Antiretroviral therapy; AU: Auranofin; BSO: Buthionine sulfoximine; H-iART: Highly intensified ART; HIV: Human immunodeficiency virus; SIV: Simian immunodeficiency virus; TCM: T central memory; TEM: T effector memory; TN: T naïve.

## Competing interests

The Istituto Superiore di Sanità has requested patent rights on the use of the auranofin/BSO therapeutic combination for treatment of HIV/AIDS.

## Authors’ contributions

AS and EG conceived the study; AS designed the experiments, drafted the manuscript, analyzed the data and coordinated the study; ILS contributed to data analysis, manuscript drafting, experimental design and project coordination, and conducted *in-vitro* experiments and *ex-vivo* immunological analyses; BC conducted *in vitro* experiments. ATP and RS measured the NF-kappaB levels and contributed the BSO administration protocol; MGL and WW conducted the *in-vivo* experiments on macaques and coordinated the related clinical analyses; LL cultivated the HIV-1 isolates employed for the *in-vitro* experiments; CL and DM performed the virus neutralization assays; MF conducted the NASBA viral load quantitation; ADC conducted the numerical simulations and contributed to data analysis and interpretation. All authors read and approved the final manuscript.

## Supplementary Material

Additional file 1 **Auranofin and BSO induce a “shock and kill” effect on monocyte-derived macrophages.** This additional file describes the *in-vitro* experiments conducted on macrophages. Click here for file

Additional file 2 **Validation of NASBA sensitivity over time.** Comparison between the input number of viral RNA copies employed as positive controls and the output value (viral RNA copies/mL) yielded by the NASBA assay for each positive control. All the positive controls shown were run in parallel with the plasma samples of macaque 4890 that yielded an undetectable viral load (*i.e.* < 50 viral RNA copies/mL). Click here for file

Additional file 3 Neutralizing antibody titers in SIVmac251-infected macaques before and after treatment with H-iART/auranofin (AU) with or without BSO. Click here for file

Additional file 4 **Numerical simulations of the Rong and Perelson model with programmed expansion and contraction of the viral reservoir.** All simulations are based on Ref. [50], including the parameters adopted for the viral burst size in macaques and humans. Panels A,B show the typical disease progression in a macaque (A) and a human (B) model. According to our previous study [12], the death rate of productively infected CD4^+^ T-cells in the macaque model is assumed to be 7.20 day^-1^. In both scenarios there is no control of the infection, and a clear increase (of about 3 *Logs*) of the latent reservoir is observed. For starting data, see Additional file 7, while, for a thorough discussion of the parameters and for the activation function used, see Additional file 6. Panels C,D illustrate a theoretical situation in which the death rate of productively infected CD4^+^ T-cells (which can be interpreted as a measure of the immune response to the virus) is increased of about tenfold. Note that CD4 counts are referred to 1 mL of blood and are expressed in the *Log* scale. To allow a visualization in *Log* scale, all the graphs have been shifted upwards (+0.1). Click here for file

Additional file 5 **Criteria for the calculation of the post-therapy viral set points in macaques subjected to different types of treatment.** This additional file illustrates the criteria employed for the calculation of the viral set points following therapy interruption. Click here for file

Additional file 6 **Mathematical modeling.** This additional file provides detailed information on the mathematical modeling procedures and rationale. Click here for file

Additional file 7 Starting data for the numerical simulation of the viral load/T-cell dynamics in SIVmac251-infected macaques. Click here for file
