# Supplementary material for: Investigational treatment suspension and enhanced cell-mediated immunity at rebound followed by drug-free remission of simian AIDS
Source: Retrovirology. 2013 Jul 16;10:71. doi: 10.1186/1742-4690-10-71 (PMC3748827; doi:10.1186/1742-4690-10-71)
Supplement: Additional file 1 — Auranofin and BSO induce a “shock and kill” effect on monocyte-derived macrophages. This additional file describes the in-vitro experiments conducted on macrophages. [file 1742-4690-10-71-S1.docx]

**Additional file 1. Auranofin and BSO induce a “shock and kill” effect on monocyte-derived macrophages.**

In a previous study, we verified that auranofin was unable to induce HIV-1 reactivation from latency in primary CD4^+^ T-cells (see below Ref. [1]). As different mechanisms are involved in virus reactivation from latency in primary CD4^+^ T-cells and macrophages (the former depending on NF-AT and the latter depending on NF-κB-mediated redox-sensitive stimuli [2,3]), we tested whether treatment with auranofin and/or BSO might change the levels of nuclear NF-κB in the human macrophage cell line U937 (Fig.1). Upon 6h treatment with auranofin (500 nM), there was a trend towards increased intranuclear levels of NF-κB (Fig. 1). The increase became statistically significant when the combination of auranofin (500 nM) and BSO (200 μM) was used (Fig.1). Prompted by this observation, we analyzed whether treatment with auranofin and/or BSO could induce HIV-1 reactivation from latency in primary monocyte-derived macrophages. Monocytes were isolated from human total blood and induced to differentiate into macrophages using GM-CSF (10 ng/mL). When the majority of cells in 96-well plates had assumed a macrophage-like morphology they were infected with R5 HIV-1 strains/isolates. The viruses adopted were ADA, BAL and J45CPpIII:2 (an isolate from the San Raffaele Scientific Institute, Milan, Italy) and were used at a concentration of 1 ng of p24/well. After overnight incubation with the virus, cells were washed three times and HIV-1 p24 production was subsequently monitored at regular intervals using a commercially available kit (INNOTEST HIV Antigen mAb, Innogenetics), until the p24 levels spontaneously decreased to the values of mock-infected controls in accordance with previous observations [4]. Macrophages were then treated with auranofin (500 nM), BSO (200 μM) and the combination of the two for 3-5 days at the same concentrations employed in the U937 experiment.

Differently from the results obtained in CD4^+^ T-cells, auranofin induced viral reactivation from latency in macrophages (Fig. 2B). Similar results on the same cells were shown by BSO (Fig. 2B), in line with previous experiments conducted in productively infected macrophages and showing a BSO-induced enhancement of viral replication [5]. The effects of auranofin and BSO on viral reactivation were not synergistic in terms of p24 levels (Fig. 2B), likely because the combination of auranofin and BSO significantly decreased the viability of infected macrophages (Fig. 2C,D). The treatments, however, did not show any significant effect on cell viability of uninfected macrophages (Fig. 2). In conclusion, the increase in nuclear NF-κB levels induced by the combination of auranofin and BSO may promote viral escape from latency in macrophages and, coupled to the oxidative stress induced by HIV infection [5] and by the treatment itself [6,7], may explain the “shock and kill” effect induced by this drug combination.

**Figure 1. Effects of auranofin (AU) and BSO on the nuclear NF-κB levels in the human macrophage cell line U937.** Panel A**.** NF-κB levels were measured after isolation of the nuclei with the nuclear and cytoplasmic extraction reagent (NE-PER) kit (Pierce). Protein loading was assessed by stripping the membrane and reprobing it with an anti-lamin antibody. Panel B. Densitometric analysis of nuclear NF-κB levels. Results are expressed as ratio over control of nuclear NF-κB to lamin. A representative value for TNF-α treated cells is shown as a positive control. Data were analyzed by repeated measures ANOVA followed by Newman-Keuls post-test.

**Figure 2. Effects of auranofin and BSO on uninfected and latently HIV-1-infected monocyte-derived macrophages.** Panels A-C show data obtained from uninfected and HIV-1 infected-macrophages. Panels A and C show the cell viability as tested by MTT assay on monocyte derived macrophages isolated from four different donors (one donor infected with HIV-1 ADA, another donor with HIV-1 J45CPpIII:2 and two donors infected with HIV-1 BAL). Panel B shows viral reactivation as tested by HIV-1 p24 ELISA (values using the ADA strain are shown). One representative experiment. Panel D shows cell viability as tested by staining with annexin V upon 24 and 48 h of treatment, (*x* axis). Data in Panels A,C were analyzed by repeated measures ANOVA followed by Newman-Keuls post-test.

**References**

1. Lewis MG, DaFonseca S, Chomont N, Palamara AT, Tardugno M, Mai A, Collins M,Wagner WL, Yalley-Ogunro J, Greenhouse J, Chirullo B, Norelli S, Garaci E, Savarino A. **Gold** **drug auranofin restricts the viral reservoir in the monkey AIDS model and induces containment of viral load following ART suspension.** *AIDS* 2011, **25**(11):1347-56.
2. Bosque A, Planelles V. **Induction of HIV-1 latency and reactivation in primary memory CD4+ T cells.** *Blood* 2009, **113**(1):58-65. doi:10.1182/blood-2008-07-168393
3. Herbein G, Varin A. **The macrophage in HIV-1 infection: from activation to deactivation?** *Retrovirology* 2010;**7**:33. doi: 10.1186/1742-4690-7-33. Review.
4. Diget EA, Zuwala K, Berg RK, Laursen RR, Søby S, Østergaard L, Melchjorsen J, Mogensen TH. **Characterization of HIV-1 infection and innate sensing in different types of primary human monocyte-derived macrophages.** Mediators Inflamm.2013; 2013:208412.
5. Garaci E, Palamara AT, Ciriolo MR, D'Agostini C, Abdel-Latif MS, Aquaro S, Lafavia E, Rotilio G. **Intracellular GSH content and HIV replication in human macrophages.** *J Leukoc Biol.* 1997;**62**(1):54-9.
6. Cox AG, Brown KK, Arner ES, Hampton MB. **The thioredoxin reductase inhibitor auranofin triggers apoptosis through a Bax/Bak-dependent process that involvesperoxiredoxin 3 oxidation.** *Biochem Pharmacol.* 2008, **76**(9):1097-109. doi:10.1016/j.bcp.2008.08.021.
7. Griffith OW. **Mechanism of action, metabolism, and toxicity of buthioninesulfoximine and its higher homologs, potent inhibitors of glutathione synthesis.** *J Biol Chem.* 1982, **257**(22):13704-12.
