# Supplementary figures and images for: Investigational treatment suspension and enhanced cell-mediated immunity at rebound followed by drug-free remission of simian AIDS
Source: Retrovirology. 2013 Jul 16;10:71. doi: 10.1186/1742-4690-10-71 (PMC3748827; doi:10.1186/1742-4690-10-71)

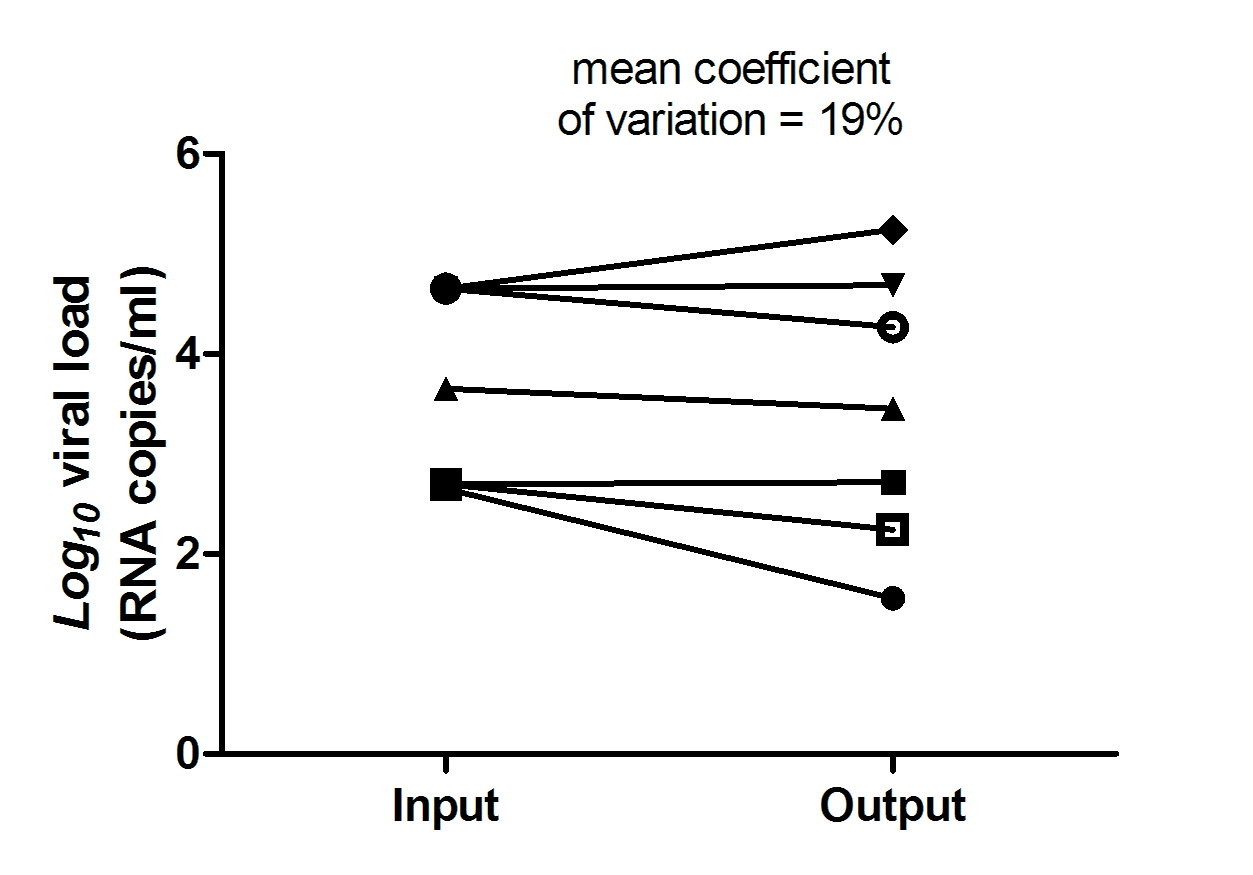

Supplement: Additional file 2 — Validation of NASBA sensitivity over time. Comparison between the input number of viral RNA copies employed as positive controls and the output value (viral RNA copies/mL) yielded by the NASBA assay for each positive control. All the positive controls shown were run in parallel with the plasma samples of macaque 4890 that yielded an undetectable viral load (i.e. < 50 viral RNA copies/mL). [file 1742-4690-10-71-S2.tiff]

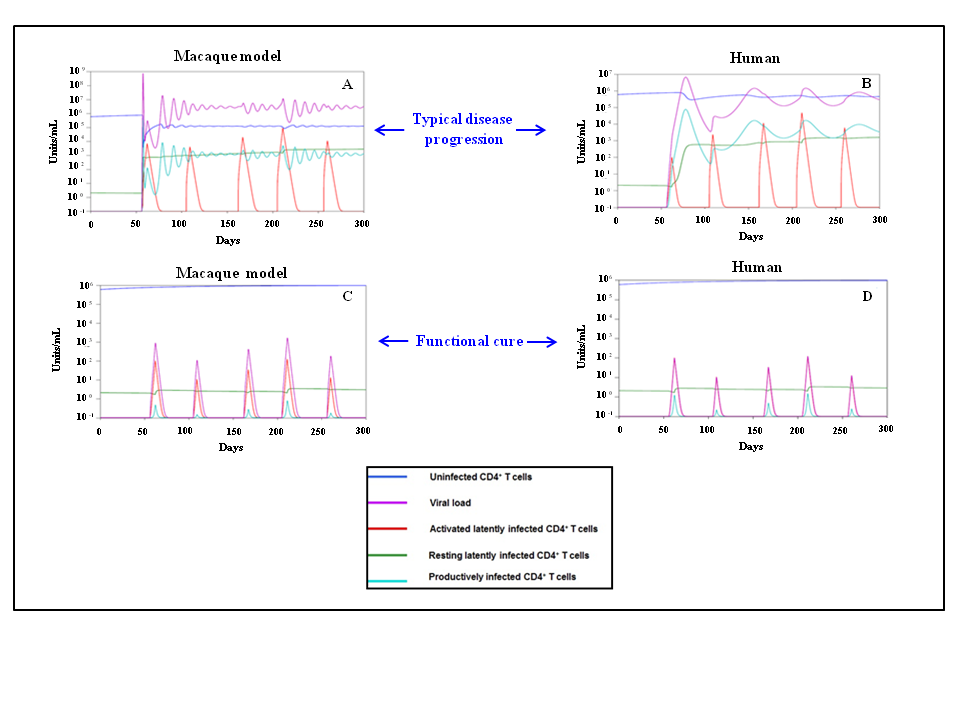

Supplement: Additional file 4 — Numerical simulations of the Rong and Perelson model with programmed expansion and contraction of the viral reservoir. All simulations are based on Ref. [50], including the parameters adopted for the viral burst size in macaques and humans. Panels A,B show the typical disease progression in a macaque (A) and a human (B) model. According to our previous study [12], the death rate of productively infected CD4+ T-cells in the macaque model is assumed to be 7.20 day-1. In both scenarios there is no control of the infection, and a clear increase (of about 3 Logs) of the latent reservoir is observed. For starting data, see Additional file 7, while, for a thorough discussion of the parameters and for the activation function used, see Additional file 6. Panels C,D illustrate a theoretical situation in which the death rate of productively infected CD4+ T-cells (which can be interpreted as a measure of the immune response to the virus) is increased of about tenfold. Note that CD4 counts are referred to 1 mL of blood and are expressed in the Log scale. To allow a visualization in Log scale, all the graphs have been shifted upwards (+0.1). [file 1742-4690-10-71-S4.tiff]
