# Supplementary material for: Investigational treatment suspension and enhanced cell-mediated immunity at rebound followed by drug-free remission of simian AIDS
Source: Retrovirology. 2013 Jul 16;10:71. doi: 10.1186/1742-4690-10-71 (PMC3748827; doi:10.1186/1742-4690-10-71)
Supplement: Additional file 3 — Neutralizing antibody titers in SIVmac251-infected macaques before and after treatment with H-iART/auranofin (AU) with or without BSO. [file 1742-4690-10-71-S3.docx]

|  |  |  |  |  |  |  |
| --- | --- | --- | --- | --- | --- | --- |
|  |  | **ID_50_ in TZM-bl Cells** | | | **ID_80_ in TZM-bl Cells** |  |
|  |  | **SVA-MLV (neg. ctrl.)** | **SIVmac251.30 Tier 2** | **SIVmac251.6 Tier 1** | **SIVmac251.6 Tier 1** |  |
| **Animal** | **Time point** |  |  |  |  |  |
| **P157** | Pre-ART | <20 | **465** | **>43740** | **230** | 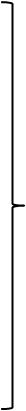   \|  \| \| --- \| |
|  | 1 month post BSO | <20 | **2532** | **>43740** | **1873** |  |
|  | 4 months post BSO | <20 | **575** | **>43740** | **379** |  |
|  | 9 months post BSO | <20 | **960** | **>43740** | **423** |  |
|  | 13 months post BSO | <20 | **497** | **>43740** | **267** | H-iART + AU + BSO |
| **P252** | Pre-ART | <20 | **186** | **>43740** | **98** |  |
|  | 1 month pre-BSO | 21 | **437** | **>43740** | **216** |  |
|  | 4 months post BSO | <20 | **297** | **>43740** | **233** |  |
|  | 6 months post BSO | <20 | **392** | **>43740** | **525** |  |
| **4890** | Pre-ART | 116 | **181** | **263519** | **118** |  |
|  | 0 months post BSO | <20 | **27** | **125616** | **30** |  |
|  | 1 month post BSO | <20 | **28** | **141166** | **<30** |  |
|  | 2 months post BSO | <20 | **100** | **544528** | **42** |  |
| **P177** | Pre-ART | <20 | **55** | **>43740** | **46** | 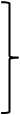  H-iART + AU |
|  | 2 months post therapy | <20 | **90** | **>43740** | **152** |  |
|  | 7 months post therapy | <20 | **128** | **>43740** | **54** |  |
|  | 11 months post therapy | 29 | **141** | **>43740** | **70** |  |
|  |  |  |  |  |  |  |
|  |  |  |  |  |  |  |
