# Supplementary material for: Investigational treatment suspension and enhanced cell-mediated immunity at rebound followed by drug-free remission of simian AIDS
Source: Retrovirology. 2013 Jul 16;10:71. doi: 10.1186/1742-4690-10-71 (PMC3748827; doi:10.1186/1742-4690-10-71)
Supplement: Additional file 5 — Criteria for the calculation of the post-therapy viral set points in macaques subjected to different types of treatment. This additional file illustrates the criteria employed for the calculation of the viral set points following therapy interruption. [file 1742-4690-10-71-S5.docx]

**Additional File 5. Criteria for the calculation of the post-therapy viral set points in macaques subjected to different types of treatment**.

1) Calculation of the post-therapy viral set point in macaques subjected to treatment with auranofin, or auranofin and BSO, and showing an acute infection-like peak in viremia at rebound. The examples refer to macaques P185 (left) and P157 (right).

2) Calculation of the post-therapy viral set point in animals that had the initial viral load peak abated artificially by H-iART. The example of macaque P177 is given.

3) Calculation of the post-therapy viral set point in animals that had received H-iART-only. The example of P188 is given.

Horizontal quotes (in black) mark the viral load set points (y axes) and the time frames on which their calculations are based (100 ± 15 days; x axes). The curves describing the post-therapy viral dynamics were obtained by non-linear regression of the data points corresponding to viral loads in time. Interpolations are shown only for visual clarity, and calculations of the viral set point were done using the original data points (see Fig. 2 in the main paper). For further details, see the Methods section (subchapter: “Statistical analyses”).
