# Supplementary material for: Investigational treatment suspension and enhanced cell-mediated immunity at rebound followed by drug-free remission of simian AIDS
Source: Retrovirology. 2013 Jul 16;10:71. doi: 10.1186/1742-4690-10-71 (PMC3748827; doi:10.1186/1742-4690-10-71)
Supplement: Additional file 6 — Mathematical modeling. This additional file provides detailed information on the mathematical modeling procedures and rationale. [file 1742-4690-10-71-S6.docx]

**Additional file 6. Mathematical modeling**

The 300 days simulations shown in Additional file 4 are based on the five-differential equation system (4) in Ref. [1] (see below). The simulations are performed without taking into account any action of antiretroviral drugs (drug efficacy parameter ε is always set to 0). The peaks in the viral load correspond to the periods of activation of latently infected CD4^+^ T-cells.

In the simulations shown in panels A and C of Additional file 4, referred to a macaque model, burst size (*i.e.:* the average number of virions produced by a single productively infected cell in 1 day) is assumed to be 55,000 RNA copies/day [2]. In panel A of Additional file 4, the death rate of productively infected cells has been set to 7.20 days^-1^ according to [3]. In panel B this death rate is set to 1 days^-1^ according to [1]. In panels C,D of the same figure, this death rate has been increased of about 1 *Log*, thus set to: 70 days^-1^ for the macaque model (panel C) and 10 days^-1^ for the human model (panel D). According to Ref. [1], proliferation rate of activated latently infected CD4^+^ T-cells is set to 1.4 days^-1^. Parameters are chosen according to Table 1 in Ref. [1]. Starting data are shown in Additional file 7 of the present manuscript.

The random activation function used in the simulations is shown in the figure below.

The simulations shown in Panels A-B of Additional file 4 are representative of a condition in which the immune system is unable to control the infection. These simulations may require some additional explanation.

In the mathematical model herein employed, one of the terms describing the source of productively infected CD4^+^ T-cells has the form $\left( 1-\eta\right)\kappa VT$ (see the fourth equation in the system (4) in Ref. [1]). In this term, $V$ and $T$ are respectively the number of RNA copies and the number of uninfected CD4+ T cells/mL of blood. The coefficient $\kappa$ represents the daily infection rate, and the coefficient $\eta$ represents the average fraction of infections resulting in latency. It follows that the model will not be accurate when $V>\frac{1}{\left( 1-\eta\right)k}$ ≈ $4,2\times{10}^{7}$ RNA copies/mL, because in this case the number of cells infected in 1 day exceeds the total of cells susceptible to infection in that moment, which has no biological meaning.

Since our purpose in panels A and B of Additional file 4 was to show the qualitative behavior of the infection (*i.e.*  the absence of control by the immune system) and not to accurately estimate the size of the first viral peak in the given conditions, our conclusions remain fully justified.

**Activation function.** The random step functions used in the simulations shown in Additional file 4 which determines the activation of resting latently infected CD4^+^ T-cells. According to [1], times between two activation periods follow a Poisson distribution with a mean of 50 days, and the length of activation periods follows a uniform distribution over an interval of 4 to 6 days.

**References**

1. Rong L, Perelson AS. **Modeling latently infected cell activation: viral and latent reservoir persistence, and viral blips in HIV-infected patients on potent therapy.** *PLoS Comput Biol.* 2009, **5**(10):e1000533
2. Chen HY, Di Mascio M, Perelson AS, Ho DD, Zhang L. **Determination of virus burst size in vivo using a single-cycle SIV in rhesus macaques.** *Proc Natl Acad Sci U S A.* 2007, **104**(48):19079-84.
3. Shytaj IL, Norelli S, Chirullo B, Della Corte A, Collins M, Yalley-Ogunro J, Greenhouse J, Iraci N, Acosta EP, Barreca ML, Lewis MG, Savarino A. **A highly intensified ART regimen induces long-term viral suppression and restriction of the viral reservoir in a simian AIDS model.** *PLoS Pathog.* 2012; **8**(6):e1002774. doi:10.1371/journal.ppat.1002774.
