# Supplementary material for: Investigational treatment suspension and enhanced cell-mediated immunity at rebound followed by drug-free remission of simian AIDS
Source: Retrovirology. 2013 Jul 16;10:71. doi: 10.1186/1742-4690-10-71 (PMC3748827; doi:10.1186/1742-4690-10-71)
Supplement: Additional file 7 — Starting data for the numerical simulation of the viral load/T-cell dynamics in SIVmac251-infected macaques. [file 1742-4690-10-71-S7.docx]

**Additional file 7. Starting data for the numerical simulation of the viral load/T-cell dynamics in SIVmac251-infected macaques.** The values are referred to a drug-free period. For the starting number of resting latently infected CD4^+^ T-cells, see Ref. [1] below.


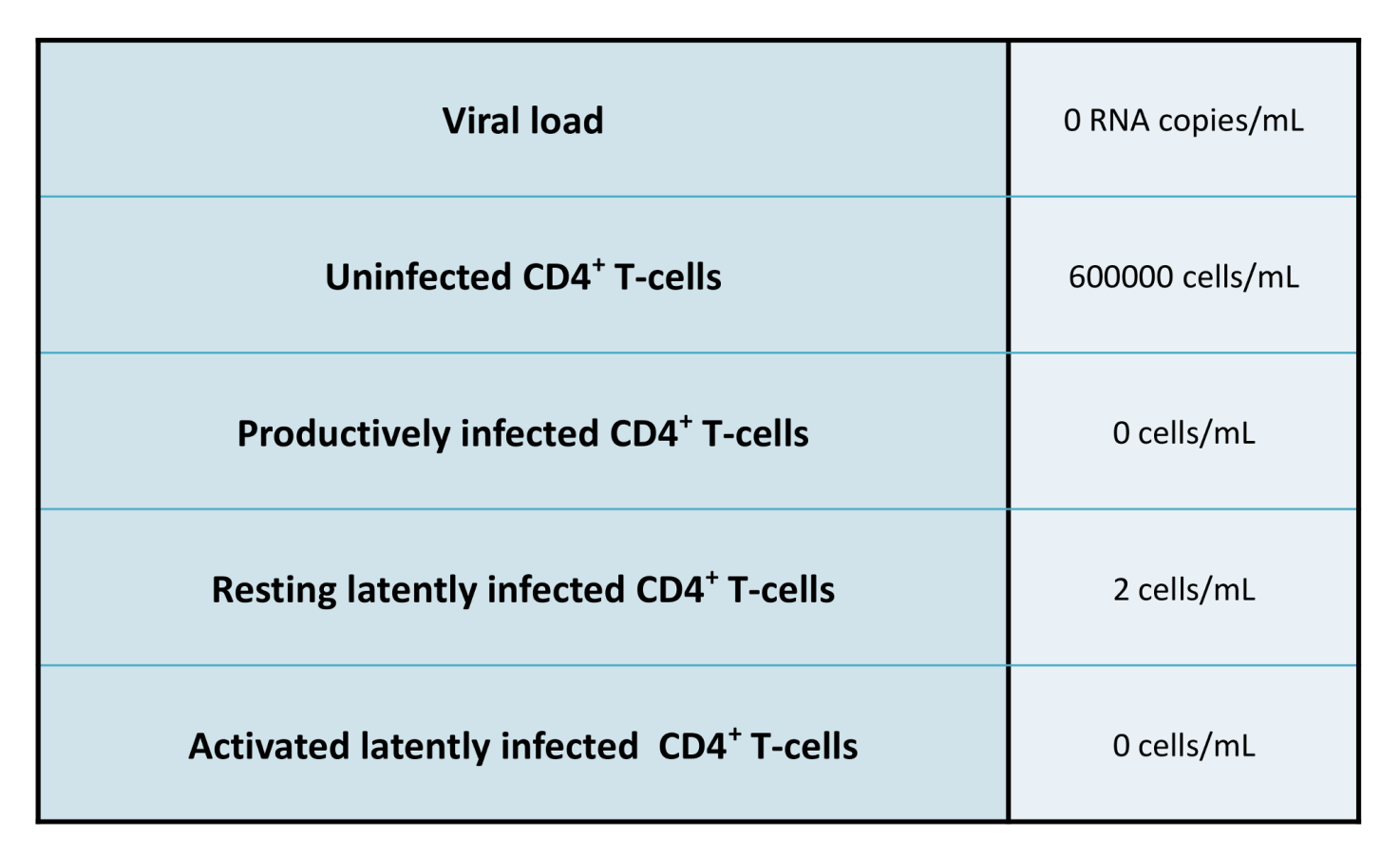


**References**

1. Shytaj IL, Norelli S, Chirullo B, Della Corte A, Collins M, Yalley-Ogunro J, Greenhouse J, Iraci N, Acosta EP, Barreca ML, Lewis MG, Savarino A. **A highly intensified ART regimen induces long-term viral suppression and restriction of the viral reservoir in a simian AIDS model.** *PLoS Pathog.* 2012; **8**(6):e1002774. doi:10.1371/journal.ppat.1002774.
